# Whole Blood Transcriptional Profiling Reveals Deregulation of Oxidative and Antioxidative Defence Genes in Myelofibrosis and Related Neoplasms. Potential Implications of Downregulation of Nrf2 for Genomic Instability and Disease Progression

**DOI:** 10.1371/journal.pone.0112786

**Published:** 2014-11-14

**Authors:** Hans Carl Hasselbalch, Mads Thomassen, Caroline Hasselbalch Riley, Lasse Kjær, Thomas Stauffer Larsen, Morten K. Jensen, Ole Weis Bjerrum, Torben A. Kruse, Vibe Skov

**Affiliations:** 1 Department of Hematology, Roskilde Hospital, University of Copenhagen, Roskilde, Denmark; 2 Department of Clinical Genetics, Odense University Hospital, Odense, Denmark; 3 Department of Hematology L, Herlev Hospital, University of Copenhagen, Herlev, Denmark; 4 Department of Hematology X, Odense University Hospital, Odense, Denmark; 5 Department of Hematology L, Rigshospitalet, University of Copenhagen, Copenhagen, Denmark; Queen's University Belfast, United Kingdom

## Abstract

The Philadelphia-negative chronic myeloproliferative neoplasms - essential thrombocythemia (ET), polycythemia vera (PV), and myelofibrosis (MF) (MPNs) - have recently been shown to be associated with chronic inflammation, oxidative stress and accumulation of reactive oxygen species (ROS). Using whole blood transcriptional profiling, we report that several oxidative stress and anti-oxidative stress genes are significantly deregulated in MPNs. Among the twenty most up- and downregulated genes, ATOX1, DEFB122, GPX8, PRDX2, PRDX6, PTGS1, and SEPP1 were progressively upregulated from ET over PV to PMF, whereas AKR1B1, CYBA, SIRT2, TTN, and UCP2 were progressively downregulated in ET, PV and PMF (all FDR <0.05). The gene Nrf2, encoding the transcription factor nuclear factor erythroid 2-related factor 2 (NFE2L2 or Nrf2) was significantly downregulated in all MPNs. Nrf2 has a key role in the regulation of the oxidative stress response and modulates both migration and retention of hematopoietic stem cells (HSCs) in their niche. The patogenetic importance of Nrf2 depletion in the context of expansion of the hematopoietic progenitor pool in MPNs is discussed with particular focus upon the implications of concomitant downregulation of Nrf2 and CXCR4 for stem cell mobilization.

## Introduction

The Philadelphia-negative chronic myeloproliferative neoplasms (MPNs) – essential thrombocythemia (ET), polycythemia vera (PV) and primary myelofibrosis (PMF) – are clonal stem cell diseases, arising due to an acquired genetic defect in the pluripotent stem cell. The nature of the initiating genetic defect remains to be established but several “second hit” genetic aberrations have been identified giving rise to dysregulation of various signaling pathways of importance in controlling blood cell production. One of these is the JAK2V617F mutation, which is present in virtually all patients with PV and in half of those with ET and PMF [1]. According to “The Biological Continuum” concept, these neoplasms evolve from an early disease stage (ET) to the advanced myelofibrosis stage, implying in the JAK2V617F-positive patients a steady increase in the JAK2V617F mutational load from “low burden” JAK2V617F-positive ET over PV to the advanced burnt-out myelofibrosis stage [1]–[3].

The MPNs are associated with a chronic inflammatory state due to the continuous release of inflammation products from in vivo activated leukocytes and platelets [4]. Indeed, the MPNs may be described as a “Human Inflammation Model”, illustrating the devastating consequences of chronic inflammation in MPNs – premature atherosclerosis, immune deregulation with loss of tumor immune surveillance, clonal evolution with myelofibrotic and leukemic transformation and an increased risk of second cancer as well [5]–[8]. Most recently the potential link between chronic inflammation and the development of myeloproliferative cancer has been described [6].

Inflammation generates reactive oxygen species (ROS), and most recently the JAK2V617F mutation per se has been shown to induce the accumulation of ROS in the hematopoietic stem cell compartment, overproduction of ROS being a mediator of JAK2V617F-induced oxidative stress, genomic instability and DNA-damage [9]. In the context of oxidative stress, the transcription factor nuclear factor erythroid 2-related factor 2 (NFE2L2 or Nrf2) has a key role in the regulation of the oxidative stress response [10]. Furthermore, most recently Nrf2 has been shown to modulate both migration and retention of hematopoietic stem cells (HSCs) in their niche, Nrf2 depletion giving rise to an expansion of the hematopoietic stem and progenitor cell (HSPC) compartment [11]. Myelofibrosis is characterized by an expansion of the HSPC pool and by egress of CD34+ positive cells from stem cell niches into the circulation to seed extramedullarily in the spleen and liver [12]. Accordingly, taken into account that chronic inflammation with ROS accumulation might induce an altered redox balance of pivotal significance for stem cell mobilization in myelofibrosis, we speculated if oxidative and anti-oxidative stress genes might be deregulated in MPNs with particular attention to the Nrf2 gene which plays such a central role in the regulation of hematopoietic stem cell (HSC) function. Using whole blood transcriptional profiling, we have identified a massive deregulation of several genes involved in oxidative stress and anti-oxidative stress mechanisms.

## Patients and Methods

Whole blood was collected from control subjects (n = 21) and patients with ET (n = 19), PV (n = 41), and PMF (n = 9) (data set 1). Patient characteristics and hematological data are shown in Table 1 and Table 2, which have been previously published [3]. Patients were diagnosed and followed in two institutions in Denmark. Most patients were studied on cytoreductive therapy, which for the large majority included hydroxyurea, being administered to 10 patients with ET, 26 patients with PV and in 1 patient with PMF (8 PMF patients received no therapy). In ET, PV and PMF patients, 9, 40 and 2 patients were JAK2V617F-positive, respectively. Whole blood from a cohort of patients with ET (n = 8), PV (n = 21) and PMF (n = 4) was collected (data set 2), compared to the control subjects (n = 21), and used to evaluate results from data set 1. Patient characteristics are shown in Table 3. Samples were collected in Paxgene tubes (Preanalytix, Hombrechtikon, Switzerland) and stored at room temperature for 24 hours, then at −20°C for minimum one day, and finally transferred to a −80°C freezer. Total RNA was extracted from each sample using the Paxgene Blood RNA kit (Qiagen, Franklin Lakes, NJ, USA). The quantity and quality of RNA were tested with NanoDrop spectrophotometer ND-8000 (NanoDrop Technologies) and Agilent 2100 Bioanalyzer (Agilent Technologies, Palo Alto, CA), respectively. The Message-AmpTM III RNA amplification kit (Ambion, Austin, TX) was applied to convert 300 ng of purified total RNA to biotin-labeled aRNA. Labeled aRNA was fragmented and hybridized to Affymetrix HGU133 Plus 2.0 chips.

**Table 1:** Patient characteristics–Data set 1.

	No	Gender (m/f)	Age (years)	Disease duration (months)	JAK2V617F (+/−)	V617F allele burden %	Therapy	Thrombosis (+/−)
ET	19	9/10	60 (35–87)	40 (15–278)	9/10	23 (1–55)	HU = 10 IFN = 3 ANA = 5 BU = 1	9/10
PV	41	21/20	69 (35–85)	39 (2–171)	40/1	37 (28–48)	None = 3 HU = 26 IFN = 5 ANA = 1 BU = 6	19/22
PMF	9	3/6	68 (53–74)	31 (11–204)	2/7	59	None = 8 HU = 1	1/8

Age: Median and range; Disease duration: Median and range; V617F allele burden %: median and 95% confidence interval. Therapy: HU  =  hydroxyurea, IFN  =  recombinant interferon-alfa, ANA  =  anagrelide; BU  =  busulfan.

**Table 2:** Hematological data–Data set 1.

	ET	PV	PMF	P-value
Hemoglobin g/dl	13.4 (11.6–14.4)	13.5 (12.9–13.7)	11.1 (9.7–11.8)	<.00001
Leucocytes ×10^9^/l	5.3 (4.2–8.1)	7.6 (6.6–8.8)	7.3 (2.3–18.7)	NS
Neutrophils ×10^9^/l	3.65 (2.31–5.63)	5.32 (4.22–6.21)	4.31 (1.10–9.82)	NS
Lymphocytes ×10^9^/l	1.60 (0.35–0.56)	1.44 (0.33–0.55)	1.06 (0.60–3.08)	NS
Monocytes ×10^9^/l	0.45 (0.35)	0.44 (0.33–0.55)	0.80 (0.17–2.31)	NS
Immatures ×10^9^/l	NA	NA	0.36 (0.0–4.81)	NA
Platelets ×10^9^/l	400 (310–519)	386 (319–450)	80 (32–232)	0.0001

Hemoglobin concentration and cell counts are presented as medians with 95% confidence intervals in parenthesis. NA: Not applicable. NS: Not significant.

**Table 3:** Patient characteristics–Data set 2.

	No	Gender (m/f)	Age (years)	JAK2V617F(+/)	V617Fallele burden (%)	Therapy
ET	8	4/4	57(45–66)	6/2	14(1–48)	HU = 5 None = 3
PV	21	10/11	62(26–69)	21/0	20(10–79)	HU = 14 None = 5 ANA = 2
PMF	4	2/2	68(55–73)	4/0	30(6–92)	None = 4

Age: Median and range; Disease duration: Median and range; V617F allele burden %: median and 95% confidence interval. HU  =  hydroxyurea, ANA  =  anagrelide.

Background correction, normalization, and gene expression index calculation of probe intensities were done in R [13] using the robust multi-array average (rma) method [14]. Only perfect match probes were used for data analysis. The regularized t-test limma [15] was applied to calculate differences in gene expression between patients and controls, and the Benjamini Hochberg method using the false discovery rate (FDR) was used to correct for multiple hypothesis testing [16]. An FDR <0.05 was considered significant. Data from both data set 1 and 2 have been deposited into Gene Expression Omnibus (http://www.ncbi.nlm.nih.gov/geo; accession no. GSE26049/GSE61629, respectively).

### Ethics statement

The study was approved by The Regional Scientific Ethical Committees for Southern Denmark and was performed in accordance with the Helsinki Declaration. All patients provided written informed consent to participate in the study.

## Results

**Figure 1:**
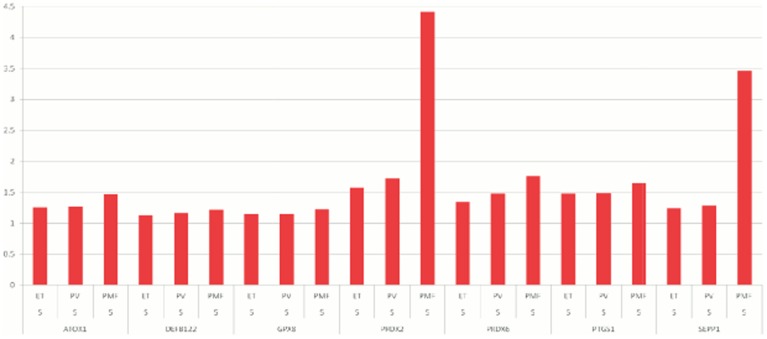
ATOX1, DEFB122, GPX8, PRDX2, PRDX6, PTGS1, and SEPP1 were progressively and significantly upregulated in patients with ET, PV, and PMF (FDR <0.05). Fold changes for each gene are shown on the y-axis.

**Figure 2:**
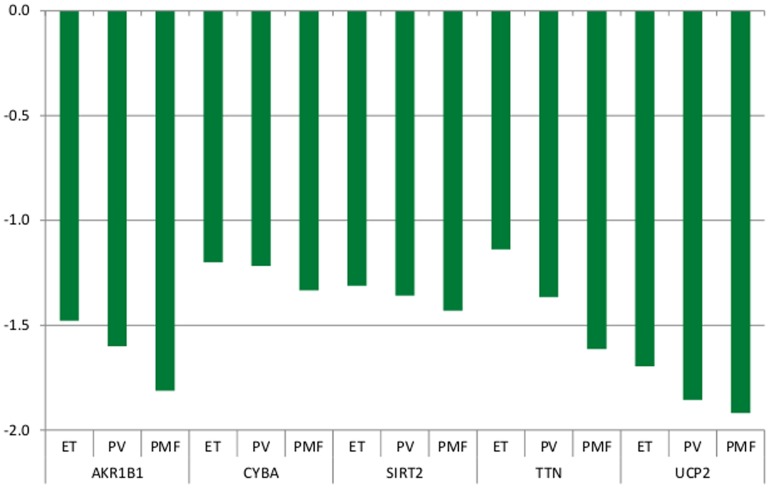
AKR1B1, CYBA, SIRT2, TTN, and UCP2 were progressively and significantly downregulated in patients with ET, PV, and PMF (FDR <0.05). Fold changes for each gene are shown on the y-axis.

**In data set 1,** 20,439, 25,307, and 17,417 probe sets were identified to be differentially expressed between controls and patients with ET, PV, and PMF, respectively (FDR <0.05). 148 of these genes were found to be included in previous studies focusing on deregulation of oxidative stress genes in various diseases and were chosen for further analysis (Table S1). 35, 40, and 46 oxidative stress genes were significantly upregulated and 33, 37, and 23 were significantly downregulated in patients with ET, PV, and PMF, respectively. The twenty most up- and downregulated genes are shown in Table 4 and Table 5, respectively. ATOX1, DEFB122, GPX8, PRDX2, PRDX6, PTGS1, and SEPP1 were progressively upregulated from ET over PV to PMF (Figure 1), whereas AKR1B1, CYBA, SIRT2, TTN, and UCP2 were progressively downregulated in ET, PV and PMF (Figure 2) (all FDR <0.05). Since inactivation of certain genes - e.g. FoxO3, TP53 and ATM - are associated with increased ROS levels and impairment of hematopoietic stem cell function, the expression of these genes was included as well. The FoxO1 gene was significantly downregulated in PV: (Fold Change (FC)  = −1.22; FDR  = 0.01) and PMF: (FC  = −2.0; FDR  = 4.4E-06). The FoxO3 gene was significantly upregulated in PV: FC  = 1.6, FDR  = 6.3E-05 and PMF: FC  = 1.7, FDR  = 0.008). The TP53 gene was highly significantly downregulated in ET, PV and PMF: (FC −1.5, −1.5 and −1.5, respectively; FDR  = 2.6E-07, 2.6E-14, and 3.4E-05, respectively. The ATM gene was significantly and progressively downregulated in ET, PV and PMF: (ET: FC = −1.3; FDR  = 0.0006; PV: FC  = −1.3, FDR  = 2.3E-06; PMF: FC  = −1.5, FDR  = 0.0002). CCND1 was highly significantly upregulated in ET, PV and PMF: FC  = 1.3, 1.3, 1.9, respectively; FDR  = 8.9E-06, 2.9E-06, 0.009, respectively; and CXCR4 was highly significantly downregulated in ET, PV and PMF: FC  = −2.1, −2.0, −2.8, respectively; FDR  = 3.7E-09, 1.1E-11, 1.5E-09, respectively. No significant differences in white blood cell counts and differential counts were recorded between the three subgroups of patients (Table 2). The 20 most up- and downregulated genes from data set 1 are evaluated in data set 2 and shown in Table 4 and 5, respectively.

**Table 4:** The top 20 most upregulated oxidative stress and antioxidative defense genes in patients with ET, PV, and PMF (FDR <0.05).

ET					PV					PMF				
	Data set 1		Data set 2			Data set 1		Data set 2			Data set 1		Data set 2	
Gene Symbol	FC	FDR	FC	Pvalue	Gene Symbol	FC	FDR	FC	Pvalue	Gene Symbol	FC	FDR	FC	Pvalue
MT2A	1.6	8.7E-05	1.4	0.001	MT2A	1.8	6.7E-07	1.5	0.0002	DEFA4	11.9	9.4E-07	5.0	4.6E-04
PRDX2	1.6	0.01	1.6	0.01	PRDX2	1.7	0.0004	1.4	0.006	MPO	5.5	5.8E-05	3.3	1.7E-05
PTGS1	1.5	0.003	1.9	5.5E-05	PTGS1	1.5	0.0001	1.6	6.6E-05	PRDX2	4.4	9.1E-06	3.2	2.4E-06
GPX1	1.4	0.002	1.5	4.2E-05	PRDX6	1.5	5.2E-05	1.5	3.5E-05	DEFA1	3.5	0.0002	1.9	0.1
PRDX6	1.3	0.03	1.6	6.3E-04	GCLM	1.5	9.2E-05	1.4	0.008	SEPP1	3.5	0.0002	2.1	0.0001
MPO	1.3	0.005	1.2	0.03	GCLC	1.4	0.008	1.5	0.002	GCLM	3.3	7.5E-07	2.0	5.6E-04
GPR156	1.3	0.0001	1.06	0.3	TXN	1.4	0.007	1.3	0.08	GCLC	2.4	0.0002	2.5	5.2E-06
SOD3	1.3	0.0003	1.1	0.1	FTH1	1.4	0.0002	1.4	3.1E-05	TXN	2.2	0.0003	1.6	0.04
LPO	1.3	0.001	−1.0	1.0	ALOX12	1.3	0.03	1.5	0.01	MGST3	1.8	1.7E-05	1.2	0.1
DUOX1	1.3	1.3E-05	1.02	0.8	GPX1	1.3	0.001	1.3	0.004	DEFT1P	1.8	0.0002	1.5	7.9E-05
SQSTM1	1.3	5.1E-05	1.08	0.1	SEPP1	1.3	0.0001	1.2	3.6E-04	GLRX	1.8	0.002	1.3	0.2
DEFB103A	1.3	0.0007	1.08	0.2	GPR156	1.3	7.0E-06	1.1	0.009	PRDX6	1.8	0.0008	2.4	3.4E-07
ATOX1	1.3	0.001	1.1	0.04	ATOX1	1.3	5.2E-06	1.2	7.0E-05	PRDX4	1.8	0.007	1.05	0.8
ADH7	1.2	0.005	1.01	0.9	LPO	1.3	1.7E-05	1.06	0.3	PRG3	1.8	0.004	1.1	0.2
SEPP1	1.2	0.0005	1.1	0.03	SQSTM1	1.2	4.6E-06	1.1	0.0002	GPX1	1.7	6.7E-05	1.7	8.0E-06
GSTZ1	1.2	0.0004	1.05	0.4	MPO	1.2	0.006	1.3	0.02	MT2A	1.7	0.0009	1.4	0.006
GPX2	1.2	0.002	1.1	0.1	ADH7	1.2	0.0004	1.04	0.3	NUDT1	1.7	3.8E-05	1.1	0.1
DEFB129	1.2	0.0009	1.2	0.001	DEFB103A	1.2	5.0E-06	1.08	0.06	PTGS1	1.6	0.02	1.8	0.009
TXNDC2	1.2	0.006	−1.0	1.0	DUOX1	1.2	3.3E-06	1.01	0.7	GSR	1.5	7.0E-06	−1.0	0.9
DEFB121	1.2	0.02	1.02	0.6	GSTZ1	1.2	4.8E-05	1.03	0.4	ATOX1	1.5	0.001	1.2	0.01

FC: fold change. FDR: false discovery rate.

The results from data set 1 are compared with results from data set 2.

**Table 5:** The top 20 most downregulated oxidative stress and antioxidative defense genes in patients with ET, PV, and PMF (FDR <0.05).

ET					PV					PMF				
	Data set 1		Data set 2			Data set 1		Data set 2			Data set 1		Data set 2	
Gene Symbol	FC	FDR	FC	Pvalue	Gene Symbol	FC	FDR	FC	Pvalue	Gene Symbol	FC	FDR	FC	Pvalue
CAT	−2.4	1.3E-08	−1.3	0.04	UCP2	−1.9	9.0E-09	−1.6	4.7E-07	PREX1	−2.6	2.3E-07	−1.6	9.2E-04
GTF2I	−1.9	4.8E-08	−1.2	0.01	CAT	−1.9	6.4E-06	−1.07	0.5	DUSP1	−2.2	0.0008	−1.6	0.01
SOD2	−1.8	0.0003	−1.4	0.1	GTF2I	−1.6	1.2E-07	−1.1	0.006	SOD2	−2.1	0.006	−1.6	0.1
HSPA1A	−1.8	0.0002	−1.04	0.7	AKR1B1	−1.6	4.4E-07	−1.4	7.5E-05	UCP2	−1.9	3.9E-06	−1.7	3.6E-05
TKT	−1.8	7.3E-06	−1.2	0.1	CYBB	−1.6	7.3E-06	−1.4	0.0001	AKR1B1	−1.8	0.0002	−1.6	9.4E-05
PREX1	−1.8	0.0002	−1.3	0.02	TKT	−1.6	2.0E-07	−1.06	0.4	PRNP	−1.7	0.002	−1.6	0.01
CYBB	−1.7	0.0003	−1.3	0.02	SOD2	−1.6	0.004	−1.5	0.008	NCF2	−1.7	4.7E-05	−1.1	0.4
UCP2	−1.7	1.8E-07	−1.3	0.02	PRPS1	−1.5	5.1E-13	−1.2	3.0E-05	GTF2I	−1.7	2.0E-05	−1.2	0.02
PGD	−1.7	0.0006	−1.02	0.9	NFE2L2	−1.5	1.5E-11	−1.2	0.0008	CCL5	−1.7	0.03	−1.3	0.2
PRNP	−1.6	0.008	−1.5	0.004	OXR1	−1.5	9.6E-09	−1.1	0.09	NCF1	−1.6	0.002	−1.1	0.5
OXR1	−1.5	4.1E-07	−1.1	0.1	GPI	−1.5	3.4E-05	−1.1	0.09	TTN	−1.6	7.8E-06	−1.3	0.008
AKR1B1	−1.5	1.5E-05	−1.2	0.02	SELS	−1.4	5.2E-06	−1.2	0.006	HSPA1A	−1.6	0.009	−1.1	0.5
CSDE1	−1.5	1.3E-05	−1.06	0.2	PREX1	−1.4	0.001	−1.1	0.1	TKT	−1.6	0.001	−1.1	0.3
PRPS1	−1.4	1.3E-09	−1.2	2.7E-04	TTN	−1.4	4.4E-06	−1.2	0.04	SIRT2	−1.4	2.6E-04	−1.2	0.05
PRDX3	−1.4	0.0004	−1.3	0.003	SIRT2	−1.4	3.2E-06	−1.02	0.7	NFE2L2	−1.4	0.001	−1.1	0.3
NFE2L2	−1.4	1.1E-05	−1.1	0.06	CSDE1	−1.3	4.8E-05	−1.09	0.1	EPHX2	−1.4	1.9E-05	−1.2	0.02
SELS	−1.4	0.0006	−1.2	0.04	CCL5	−1.3	0.03	−1.2	0.2	GSTM4	−1.4	0.02	1.1	0.4
TALDO1	−1.3	0.009	1.03	0.7	PRNP	−1.3	0.04	−1.4	0.01	ALDOA	−1.4	0.01	−1.2	0.07
GPI	−1.3	0.004	1.03	0.7	EPHX2	−1.3	8.3E-07	−1.1	0.06	CYBA	−1.3	2.6E-06	−1.0	1.0
EPHX2	−1.3	6.0E-06	−1.07	0.3	GSTP1	−1.3	0.003	−1.3	0.003	OXR1	−1.3	0.03	−1.1	0.4

FC: fold change. FDR: false discovery rate.

The results from data set 1 are compared with results from data set 2.

## Discussion

Myelofibrosis and related neoplasms are associated with a marked increase in several inflammatory cytokines [7]. Likewise, whole blood transcriptional profiling studies have unraveled a massive deregulation of inflammation and immune genes, which may be of crucial importance for disease progression [3], [17], [18]. Thus, chronic inflammation has been proposed to be both a trigger and a driver of clonal evolution, premature atherosclerosis and second cancer in MPNs [5]. Furthermore, it has been suggested that MPNs depict “A Human Inflammation Model for Cancer Development”, chronic inflammation being the driving force from early cancer stage (ET) over PV (in the JAK2V617F- positive patients) to the advanced myelofibrotic cancer stage [6].

Several biological processes are deeply dependent upon appropriate intracellular levels of ROS, including e.g. those processes being involved in the activation of signaling pathways in response to cytokines and the gene expression elicited by this signaling. However, oxidative stress may occur if ROS is produced in excessive amounts and/or if the cell's normal antioxidant defence system is defective. Chronic inflammation is associated with the generation of ROS which may ultimately give rise to oxidative stress, genomic instability, DNA-damage, and risk of mutations with the development or progression of cancer [19], [20]. A prerequisite in the defence against clonal evolution and cancer development during chronic inflammation is an effective DNA repair mechanism of the sustained oxidative stress induced by the chronic inflammatory drive. Accordingly, mutations in DNA repair mechanisms may likely increase the risk of clonal evolution in MPNs as well.

The importance of ROS for HSC function and integrity of the stem cell niche is being increasingly recognized [21], [22], and recently the link between oxidative DNA damage, genomic instability and leukemogenesis has been reviewed [23], [24]. Most recently, it has been hypothesized that the acquired stem cell lesion in MPNs might arise due to a chronic sustained inflammation stimulus being further enhanced in a selfperpetuating vicious circle by the malignant clone, which per se continuously generates inflammatory products in the bone marrow [5]–[7]. These products – e.g. tumor necrosis factor alpha (TNF-alpha) – further stimulate clonal expansion [25], [26] thus creating a positive feedback loop and a vicious circle [5]–[7]. The sustained release of inflammatory products with ensuing chronic oxidative stress due to elevated levels of ROS in the bone marrow might likely create a high-risk microenvironment for induction of oxidative damage to DNA in hematopoietic cells and mutations [6].

By whole blood transcriptional profiling we herein for the first time demonstrate that MPNs are associated with a significant upregulation of several oxidative stress genes in concert with downregulation of important antioxidative defence genes, among others a significant downregulation of the Nrf2 gene – the master regulator of the antioxidant response ensuring that chronic inflammation in normal cells slowly ceases [10]. The Nrf2 gene was significantly downregulated across all three disease categories – ET, PV and PMF. Under normal conditions, chronic inflammation is dampened due to IL-1beta induced activation of Nrf2, which subsequently activates several antioxidant genes of crucial importance for protection of cells against oxidative stress. Thus, increased Nrf2 expression up-regulates antioxidant response element (ARE)-dependent genes causing increased ARE-transcriptional activity, thereby augmenting expression of several ARE-dependent antioxidant and cytoprotective enzymes [10].

In addition to being the master regulator of the antioxidant response, Nrf2 also has a major role for normal stem cell function and is required for HSPC survival and myeloid development [11]. The role of Nrf2 in this context has been found to be independent of ROS [27]. In regard to oxidative stress and stem cell function, levels of ROS have been shown to regulate normal hematopoietic stem cells [28], implying high levels to be associated with stem cell dysfunction [29]. Lower levels in hematopoietic stem cells may explain their sustained self-renewal potential by inhibiting differentiation [30]. Thus, the marked downregulation of Nrf2 in MPNs may have a major impact upon stem cell function and behavior for several reasons. First, Nrf2 deficiency may likely compromise the antioxidant defence mechanisms against increased oxidative stress and accumulation of ROS, being induced by the inflammatory products generated by the myeloproliferation per se [5]–[7] and in the JAK2V617F positive patients also being generated by the JAK2V617F-mutation per se [9]. By an impaired defence against the increased oxidative stress in the bone marrow, accumulation of ROS may favour genotoxic damage to hematopoietic cells and stromal cells as well thereby enhancing the risk of triggering mutations [6], [7]. In this setting, increased intracellular ROS levels may also induce activation of redox-sensitive transcription factors and thereby enhancing clonal evolution and progression [31]. Second, most recently, it has been demonstrated that Nrf2 deficiency also results in an expansion of the hematopoietic stem and progenitor cell pool and a disturbed differentiation [11] which may likely contribute to enhanced clonal myeloproliferation and defective differentiation in myelofibrosis (immature red and white blood cells in the peripheral blood, respectively). Third, taking into account that Nrf2 functions as a negative regulator of cell-cycle entry in HSCs, maintaining the delicate balance between HSC quiescence and self-renewal [11] and Nrf2 also has a key role in governing the retention of HSCs and their homing to the bone marrow niche [11], Nrf2 deficiency in MPNs may likely contribute to the egress of CD34+ cells from bone marrow niches to seed preferentially in the spleen and liver (myelofibrosis with myeloid metaplasia). Of note, in regard to Nrf2 being a negative regulator of cell-cycle entry in HSCs, Nrf2 deficiency might be anticipated to be associated with higher levels of cyclin D (being synthesized during G1 phase and required for the transition to S-phase) which was also recorded in the study by Tsai et al. [11]. Importantly, we found that the cyclin D gene (CCND1) was significantly upregulated in MPNs, being most deregulated in myelofibrosis similar to Nrf2. In regard to the role of Nrf2 for efficient homing of bone marrow cells and in maintaining HSC quiescence, it has most recently been shown that Nrf2 exerts its influence, at least in part, through direct regulation of the expression of the chemokine CXCR4 which was found to be significantly reduced in HSCPs in Nrf2 deficient mice [11]. In addition, it was shown that Nrf2 directly binds to the CXCR4 promoter and activates its expression [11] explaining the dysregulation of CXCR4 in Nrf2 deficient mice. The CXCR4 chemokine has a well-established major role for HSCP homing and retention [32] but recently there has been considerable interest in CXCR4 signalling for its role in maintaining HSC quiescence as well [33], [34]. Highly interesting, CXCR4 was significantly downregulated across all three MPN categories being most pronounced in patients with myelofibrosis. This observation is consistent with previous studies displaying an altered SDF-1/CXCR4 axis with downregulation of CXCR4 in the CD34+ cells in myelofibrosis [35], [36]. Accordingly, it is intriguing to consider if the significant downregulation of Nrf2 in MPNs may not only have a profound impact upon the balance between stem cell quiescence and proliferation, between self-renewal and differentiation, but also significantly influencing homing and retention of HSCs in the bone marrow niche. Fourth, defective antioxidative defence mechanisms with excessive ROS accumulation and oxidative stress – consequent to among others Nrf2 deficiency – may likely also contribute to aberrant DNA methylation [37], which has been reported in patients with MPNs [38], including hypermethylation of the CXCR4 promoter in CD34+ cells from patients with primary myelofibrosis.

As noted above, chronic inflammation has been suggested to have a major role for disease progression in MPNs – likely being a mediator of premature atherosclerosis (and premature ageing?), clonal evolution and myelofibrotic and leukemic transformation [5]–[7]. In this context, the impact of TNF-alpha as a tumor promoter [39] and its ability to facilitate clonal expansion of JAK2V617-positive cells is of crucial importance [25], since the JAK2V617F mutation per se has most recently been shown to induce excessive ROS accumulation and accordingly oxidative stress, DNA strand breaks and mutations [9]. Furthermore, oxidative stress is associated with overproduction of several proinflammatory cytokines (e.g. TNF-alpha, IL-1beta, IL-2, IL-6, IL-8, IL-12), when the redox-sensitive NF-kappabeta is activated by oxidative stress. These cytokines, in turn, can cause oxidative stress in HSC and circulating hematopoietic cells. Considering the MPNs as “A Human Inflammation Model” with a selfperpetuating vicious circle [6] being fueled by inflammation products and excessive ROS accumulation, implying an imbalance between NF-E2 and Nrf2 [40], it is evident that this vicious circle (chronic inflammation – NF-kappa-beta-activation – production of inflammatory cytokines – ROS-generation – oxidative stress – genomic instability – clonal evolution – chronic inflammation) is only disrupted by dampening one of its very important driving forces - chronic inflammation [5]–[7]. This novel concept on pathogenesis of MPNs also dictates early upfront intervention with agents having the potential of blocking the vicious circle – interferon-alpha2 (IFN-alpha2), statins and JAK2-inhibitor treatment being potential candidates [5]–[7], [41] – both by inhibiting clonal myeloproliferation but also by impairment of ROS generation due to their very potent anti-inflammatory capacities [5]–[7]. To this end, oxidative stress and inflammation impairs IFN-alpha signaling [42] which may likely cause resistance to the effects of IFN-alpha in MPNs, including the effects of IFN-alpha2 on immune cells [43].

Inactivation of several genes – e.g. transcription factor p53 and FoxO3 – has been shown to increase ROS levels and to be associated with the loss of hematopoietic stem cell function [44], [45]. Of note, we found that the tumor suppressor p53 gene – TP53 – was significantly and progressively downregulated across all three disease categories (ET, PV and PMF). The TP53 gene has a key role in protecting the genome from oxidation by ROS with ensuing DNA damage and genetic instability. Thus, the highly significant downregulation of TP53 in our patients may result in excessive oxidation of DNA, increased mutation rate and karyotype instability [44]. The FoxO3 gene decreases ROS levels by influencing the regulation of the ataxia telangiectasia mutated (ATM) gene [34], [46], which also has a crucial role in mediating the cellular response to DNA and oxidative damage. Accordingly, downregulation of the ATM gene might profoundly enhance genomic instability consequent to increased ROS levels. In our MPN patients, the ATM gene was significantly and progressively downregulated from ET over PV to PMF, which – together with downregulation of TP53 - may have a profound negative impact upon DNA-stability. The downregulation of ATM was not associated with a downregulation of FoxO3 – actually significantly increased in our patients with PV and PMF – which accordingly may not be the main regulator of the ATM gene in our patients.

The genes UCP2 and SIRT2 were significantly and progressively downregulated in ET, PV and PMF. Both genes are involved in the regulation of NF-kappabeta, implying constitutively increased NF-kappabeta activity and production of inflammatory cytokines when these genes are deactivated or downregulated [47]. In addition, sirtuins have been described as major players in sensing and coordinating oxidative stress response and having an essential role in promoting DNA repair, telomere stability and in stem cell differentiation as well [47]. Thus, some sirtuins, such as SirT2 and SirT6, seem to work as tumor suppressors. We have previously reported significant downregulation of several sirtuins in MPNs, including – in addition to SirT2 and SirT6 – SirT3 and SirT7 [48]. Accordingly, downregulation of these genes may further prime for enhancement of inflammation, oxidative stress and genomic instability in MPNs.

As underscored above, oxidative stress induces activation of several signaling pathways including PI3K/AKT which may likely participate in the development of MPNs [49]. AKT negatively regulates the Forkhead O transcription factor family (FoxO) which targets expression of several anti-oxidant enzymes, including the GPx, catalase (CAT) and SODs. The FoxO3 gene was significantly upregulated in all three MPNs and several GPx as well. Regarding SODs, SOD2 was significantly downregulated across all three MPNs. Interestingly, the CAT gene was highly significantly downregulated in our patients with ET and PV but not in myelofibrosis. The absence of a significant downregulation of CAT in PMF was surprising, but might be related to the fact that only 2 out 9 patients were JAK2V617F-positive. Thus, most recently ROS accumulation in JAK2V617F-positive cells has been shown to be associated with a significant decrease in gene expression of CAT - both in a mouse model and in CD34+ cells from PV and myelofibrosis patients [9].

Our study has limitations and strengths. First, in the interpretation of the different genes being deregulated, it should be taken into account that our patients were not newly diagnosed but investigated at different time points after diagnosis. Second, many patients were treated with hydroxyurea (HU) at the time of investigation, which potentially might have impacted the “global signature” of increasing genomic instability (e.g. evidenced by significant downregulation of Nrf2, TP53 and ATM genes). Third, in data set 2, Nrf2 was significantly downregulated in patients with PV, showed a tendency towards downregulation in ET (P = 0.06), and was not significantly downregulated in PMF. The lack of significance in the PMF group may be due to the limited number of patients (n = 4). Fourth, we used whole blood instead of e.g. isolated granulocytes, CD34+ cells and mononuclear cells, which have been used in many previous transcriptional profiling studies in MPNs. In the context of describing “the signature of oxidative stress in haematopoietic cells”, we believe that a signature of oxidative stress and antioxidative defence genes may be more reliably obtained from transcriptional profiling of whole blood than if isolated cell types were analysed. Indeed, our previous studies of whole blood transcriptional profiling are strongly supportive, since these studies – in addition to confirming gene signatures obtained by analyzing single cells by others but generally with much stronger signals [3], [17], [18], [48], [50], [51] – also have unravelled deregulation of several genes which may be involved in disease pathogenesis and progression [3], [17], [18], [50], [51]. Our approach certainly also makes sense when considering that the “tumor tissue” being studied – whole blood – is composed of clonal “tumor” cells (being both myeloid cells, platelets, B-cells and T-cells) and non-clonal cells, including immune cells, which may also be activated due to oxidative stress fueled by clonal myeloproliferation. In this setting, whole blood transcriptional profiling is actually very similar to all other studies of gene signatures in tumor tissue. Accordingly, in the context of studying gene signatures of inflammation and oxidative stress/antioxidative defence genes in MPNs, our approach with whole blood transcriptional profiling is more a strength than a limitation. To this end, whole blood has also been used successfully in a most recent study of DNA-methylation profiles in MPNs, arguing unfractionated whole blood to be suitable for analysis [38]. Fifth, although several ROS-related genes displayed a gradual deregulation from ET, PV to PMF, we cannot definitely conclude that these changes are linked to disease progression, since the cohort did not include matched samples with identical timepoints during the course of the disease. Prospective studies of larger cohorts with serial gene expression profiling studies are needed to confirm our observations.

In conclusion, our results highlight the MPNs as novel disease categories to be listed as “Oxyradical Overload Disorders” and thereby potentially being at a risk of premature atherosclerosis and ageing in addition to the inherent risk of clonal evolution, genomic instability, leukemic transformation, and development of second cancer as well. Since chronic inflammation is likely an important trigger and driver of oxidative stress contributing to “oxyradical overload” in MPNs, this study substantiates the urgent need for early upfront interruption with agents having the potential to stop the delivery of “fuel to the fire”, implying potent anti-inflammatory and antiproliferative capacities. These agents include among others IFN-alpha2 and JAK2-inhibitors, which as monotherapies have been very promising with induction of deep and sustained molecular remissions in ET and PV patients, even after discontinuation of therapy (IFN-alpha2), rapid resolution of huge splenomegaly and constitutional symptoms (JAK2-inhibitor) and also resolution of bone marrow fibrosis after long term treatment (IFN-alpha2 and JAK2-inhibitor). In the context of dampening inflammation and oxidative stress, statins may also be highly efficacious - likely in a combinatorial approach -, since statins have been demonstrated to impair MPN cell growth and enhancing the effect of JAK2-inhibition as well [5]–[7].

## Supporting Information

Table S1 **Fold change (FC) and false discovery rate (FDR) of 148 oxidative stress genes.**
(XLS)
