# Correction: Whole Blood Transcriptional Profiling Reveals Deregulation of Oxidative and Antioxidative Defence Genes in Myelofibrosis and Related Neoplasms. Potential Implications of Downregulation of Nrf2 for Genomic Instability and Disease Progression

**DOI:** 10.1371/journal.pone.0118049

**Published:** 2015-02-02

**Authors:** The PLOS ONE Staff

The image quality of Fig. 1 is poor, making interpretation difficult. Please see the correct Fig. 1 here.

**Figure 1:**
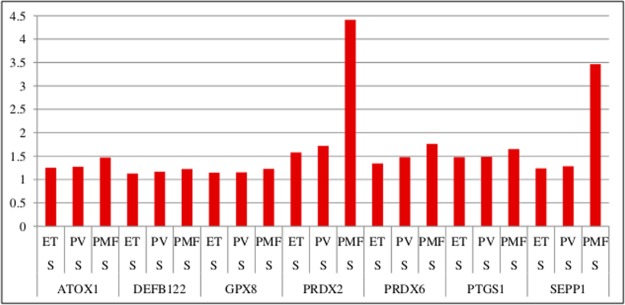
ATOX1, DEFB122, GPX8, PRDX2, PRDX6, PTGS1, and SEPP1 were progressively and significantly upregulated in patients with ET, PV, and PMF (FDR <0.05). Fold changes for each gene are shown on the y-axis. doi:10.1371/journal.pone.0112786.g001
